# Molecular basis for Gβγ-SNARE-mediated inhibition of synaptic vesicle fusion

**DOI:** 10.1016/j.jbc.2025.110377

**Published:** 2025-06-14

**Authors:** Anna R. Eitel, Benjamin K. Mueller, Ali I. Kaya, Montana Young, Jackson B. Cassada, Eric W. Bell, Lauren Schnitkey, Zack Zurawski, Yun Y. Yim, Qiangjun Zhou, Jens Meiler, Heidi E. Hamm

**Affiliations:** 1 Department of Biochemistry, Vanderbilt University, Nashville, Tennessee, USA; 2 Department of Chemistry, Vanderbilt University, Nashville, Tennessee, USA; 3 Department of Pharmacology, Vanderbilt University, Nashville, Tennessee, USA; 4 Center for Structural Biology, Department of Cell and Developmental Biology, Vanderbilt Brain Institute, Vanderbilt Kennedy Center, Nashville, Tennessee, USA; 5 Institute for Drug Development, Leipzig University, Leipzig, Germany

**Keywords:** G-protein, Gβγ subunit, SNARE protein, SNAP25, exocytosis, protein-protein interaction, neurotransmission, signal transduction, membrane fusion, GPCR signaling, protein structure

## Abstract

Neurotransmitter release is a complex process involving tightly controlled co-factors and protein-protein interactions. G-protein coupled receptors negatively regulate exocytosis *via* the interaction of G-protein βγ (Gβγ) heterodimers with the soluble N-ethylmaleimide-sensitive factor attachment protein receptor (SNARE) complex. The neuronal ternary SNARE complex comprises synaptosomal-associated protein-25 (SNAP25), syntaxin-1A, and synaptobrevin-2. The regions of the SNARE complex that are important for interactions with Gβγ have been extensively characterized, but the critical sites on Gβγ are not well understood. Furthermore, the molecular basis for the specificity of different Gβ and Gγ isoforms for SNARE proteins remains elusive. Thus, we holistically probed the entire family of human Gβ and Gγ isoforms for regions critical for the target-SNARE (tSNARE) interaction using a peptide screening approach. Gβ and γ peptides with high affinities for tSNARE were then subjected to alanine scanning mutagenesis to identify the interaction sites. We found that the N-terminal coiled-coil domain of Gβγ as well as the β-propeller domain of Gβ are hotspots for SNARE interactions. Additionally, we found that the N-terminal Gγ2 peptide is a potent inhibitor of interactions between full-length Gβ1γ2 and SNAP25. We discovered that Gβ1γ2 preferentially interacts with ternary SNARE in the pre-fusion, partially zipped conformation, likely due to increased exposure of the C-terminus of SNAP25. Our combined results suggest that specific Gβγ heterodimers bind to ternary SNARE in the docked and primed state *via* critical residues of the β-propeller and N-terminal coil-coil domains. We propose that Gβγ binding disrupts zippering up of the SNARE complex and thereby vesicle fusion.

Neurotransmitter release is a complex, regulated process involving exocytotic machinery proteins, synaptic proteins that play roles in docking and priming the vesicle, as well as ion channels, calcium sensors, and various G-protein coupled receptors (GPCRs) (1, 2, 3, 4). Understanding the molecular composition and function within the synapse has been the focus of extensive study. However, important details of the modulation of exocytosis at each step by various regulatory proteins remain unclear.

Presynaptic G_i/o_-coupled GPCRs are dominant mediators of inhibition of exocytosis in the central nervous system. Upon activation by GPCRs and liberation from the G-protein α-subunit (Gα), G-protein βγ subunits (Gβγ) mediate fast, membrane-delimited negative effects on neurotransmission, through both presynaptic voltage-gated calcium channels (VGCC) with modulation of calcium entry (5, 6), and postsynaptic activation of G-protein coupled inward-rectifying potassium (GIRK) channels (7, 8). In addition, Gβγ interacts directly with the exocytotic SNARE complex downstream of Ca^2+^ influx to inhibit synaptic vesicle fusion (1, 9, 10, 11). The interaction of Gβγ with the SNARE complex places inhibitory regulation of exocytosis at the final step of exocytotic fusion, after vesicle docking and priming (11, 12).

Five different Gβ and 12 Gγ isoforms are expressed in humans (13, 14, 15). Gβ1-4 share up to 90% amino acid sequence identity, whereas Gβ5 is only 50% identical (16, 17). In contrast, Gγ subunits share only 30 to 70% sequence identity (16, 17). Gβ subunits are made up of an α-helix of approximately 20 amino acids (H1, red, Fig. 1, *A* and *B*) and 7 blades of four anti-parallel β-strands (B1-B7, Fig. 1, *A* and *B*), a β-propeller-like WD repeat (7, 17, 18, 19, 20). A linker (L1, orange) connects the N-terminal α-helix to the β-propeller domain of Gβ (Fig. 1, *A* and *B*).

**Figure 1:**
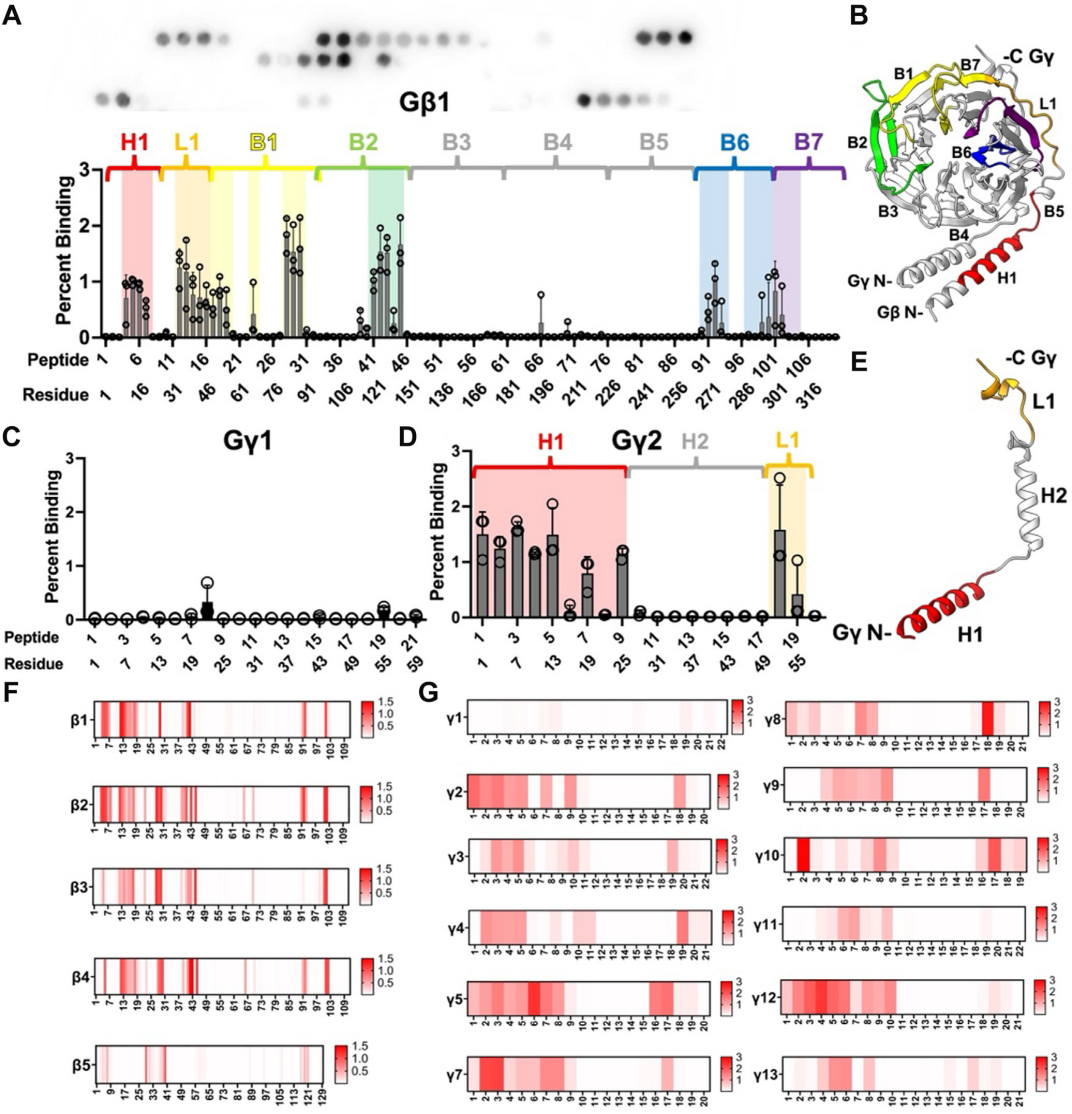
**Mapping tSNARE interactions with individual human G**β** and G**γ** isoforms using peptide array analysis.***A*, representative image of a developed membrane conjugated to Gβ1-derived peptides (bait) and quantification of peptide binding to purified tSNARE protein (prey). The sum of the densitometric signal contributed by all peptide spots on the membrane was used to define 100% binding. The binding contributed by each peptide spot to the sum was used to quantify percent binding. *Bar graphs* represent the means ± SD of three technical replicates and individual data points are superimposed. Colors correspond to regions of the Gβ1 structure. *B*, peptides that bind tSNARE are shown on the crystal structure of Gβ1γ2 (PDB: 6CRK, (45)). Gβ1 peptides that showed a significant interaction with tSNARE are colored as in A. *C*, quantified binding of tSNARE to Gγ1 and (*D*) Gγ2 peptides. *E*, peptides from Gγ2 that interacted with tSNARE mapped onto the Gβ1γ2 crystal structure with Gβ1 removed for clarity. *F*, Heat maps using a single gradient to display the ranges of binding between tSNARE and Gβ1-5 and (*G*) Gγ1-13 calculated using GraphPad Prism. A 15-mer glycine peptide was used to determine non-specific interactions. Human SNAP25 peptides between amino acids 91 to 105 and 106 to 121 were used as a positive control for the SNAP25 primary antibody.

Gγ subunits contain an N-terminal α-helix (H1, red) which forms a coiled-coil interaction with the N-terminal helix of Gβ (Fig. 1, *D* and *E*). The C-terminal α-helix (H2, gray) of Gγ interacts with the surface of blade 5 and a small section of the N-terminal region on Gβ (18, 19, 20). Gγ subunits can be post-translationally modified at the processed C-terminal cysteine, which is carboxymethylated and modified with a farnesyl or geranylgeranyl moiety *via* a thioether bond. These modifications mediate membrane localization of Gβγ (21, 22). Together, Gβ and Gγ subunits form Gβγ heterodimers, and once assembled, act as signaling units for GPCRs and modulate synaptic transmission by interacting with many effectors such as VGCC and the SNARE complex to regulate neurotransmitter release at the synapse, as well as GIRK channels (5, 9, 11, 12, 23, 24, 25, 26).

SNAP25 is the most important Gβγ partner in the interaction with ternary SNARE, a trimer comprised of SNAP25, syntaxin-1A, and the vesicle-SNARE synaptobrevin-2 (VAMP2), as well as with target-SNARE (tSNARE), a dimer comprised of SNAP25 and syntaxin-1A (9, 10, 11, 26, 27, 28, 29, 30). Multiple studies have demonstrated that the C-terminus of SNAP25 is essential for both the Gβγ-SNARE interaction and Gβγ-SNARE-mediated inhibition of exocytosis. Electrophysiological studies of the lamprey giant synapse revealed that presynaptic injection of botulinum toxin A (BoNT/A), which cleaves the nine C-terminal amino acids of SNAP25, abolished Gβγ-SNARE-mediated inhibition of evoked excitatory post-synaptic currents (EPSCs). Injection of the 14 amino acid peptide corresponding to the C-terminus of SNAP25 had the same effect (9, 11, 26, 30, 31, 32).

*In vitro* binding studies have demonstrated direct Gβγ-interaction sites on SNAP25 at both the vesicle proximal C-terminus as well as the amino-terminal end of the SNARE complex where zipping up begins (12, 33). Asp99 and Lys102 in the linker region between the two helices of SNAP25, near its palmitoylation sites (Cys85, Cys88, Cys90, and Cys92), were also found to be important for the interaction with Gβγ (12, 26). Interestingly, Gly63 and Met64 that showed a significant reduction in binding when substituted for alanine, are buried in the interface with syntaxin-1A and synaptobrevin-2 (VAMP2). In addition, the H_3_ domain of syntaxin-1A alone also interacts with Gβγ (26).

Numerous studies probed the sites on the SNARE complex of Gβγ interaction, but it is still unclear where the interaction sites are on Gβγ. The Gβγ-effector interaction sites were hypothesized to be localized at the Gα interface (34). However, mutagenesis studies revealed the binding regions of effectors within the blades of the Gβ propeller and its N-terminal coiled-coil, the Gα subunit interface, and regions on the Gγ subunit (17, 19, 35, 36, 37). For example, GIRK channels interact just outside of the Gα binding site. Thr86, Thr87, and Gly131 of Gβ1 were shown to be important for the Gβγ-GIRK channel interaction (38).

The Gα interacting residues that were shown to be important for Gβγ interactions with GIRK, VGCC, phospholipase C beta (PLCβ), G-protein coupled receptor kinase-2 (GRK2), and Gα (34), were shown to be inhibitory for interaction with the SNARE complex and inhibition of exocytosis. Unexpectedly, Ala mutations of several Gα interface residues increased the affinity of Gβγ with the SNARE complex and made the inhibition of exocytosis more potent (10). Ala substitution of both Gβ1 Lys78 and Trp332 doubled the affinity of Gβ1γ2 for tSNARE (39). While the Gα-Gβγ interface likely represents a core site of Gβγ-effector interactions, other regions may also play important roles in facilitating Gβγ-mediated downstream signaling.

We have recently reported the specificity of Gβγ interactions with the α_2A_ adrenergic receptor (α_2A_AR) and the SNARE complex based on proteomic studies (40). Here, we address the molecular basis of the specificity of different Gβ and Gγ isoforms for the tSNARE complex using peptide arrays. We identified tSNARE binding hotspots on Gβγ and determined the residues of Gβ1γ2 that are critical for the interaction. Peptides identified in our array are capable of disrupting interactions between full-length Gβ1γ2 and SNAP25, indicating that they correspond to regions of Gβγ that are important for the protein-protein interaction. Our results suggest that there are two major sites of interaction on Gβ1γ2 for tSNARE, one involving the N-terminal coiled-coil motif and one involving the β-propeller. Furthermore, we found that Gβ1γ2 preferentially interacts with a pre-fusion, partially zipped ternary SNARE mimetic (41) as opposed to the fully zipped mimetic (42, 43). Based on these results, we propose a molecular mechanism in which specific Gβγ heterodimers stabilize the disordered C-terminus of the partially zipped SNARE complex and disrupt SNARE complex zippering, thereby preventing vesicle fusion.

## Results

### Molecular mapping of tSNARE interactions with human G**β** and G**γ** isoforms

To characterize the SNARE binding sites on G-protein β and γ subunits, we used a peptide array assay (44). Peptides corresponding to the sequences of human Gβ and Gγ proteins were synthesized as previously described (44). The spots on the membrane consisted of 15-mer peptides, shifting three amino acids for each successive peptide, which cumulatively represent the entire sequence of the proteins. Wild-type tSNARE (SNAP25/syntaxin-1A dimer) was co-expressed and purified as described previously (12). Membranes were incubated with purified tSNARE and probed for SNAP25 using a far Western blot.

Figure 1*A* shows a representative blot and quantification of the interaction between Gβ1 peptides and tSNARE. The binding of each peptide spot was expressed as a percentage of total density detected on the membrane (Fig. 1*A*). Bar graphs represent the mean and SD of three experiments. Peptides that bound to tSNARE were mapped onto the Gβ1γ2 crystal structure and colored by region (Fig. 1*B*, PBD: 6CRK) (45). The Gβ1 N-terminal helix (H1) is colored red, the loop between the N-terminal coiled-coil domain and the β-propeller domain (L1) is colored orange, Blades 1 to 2 of the β-propeller domain (B1 and B2) are colored yellow and green, and Blades 6 to 7 (B6 and B7) are colored blue and purple, respectively.

We used the same approach to detect interacting regions of individual Gγ isoforms with tSNARE. The amino acid sequences for Gγ isomers are significantly less conserved than Gβ isoforms. Therefore, we expected to see more variability in binding patterns with Gγ isoforms. Figure 1, *C* and *D* show binding between Gγ1 and Gγ2 peptides and tSNARE. The data clearly indicate that tSNARE binding is considerably higher to Gγ2 peptides than to Gγ1_._ This is consistent with previous studies that found that Gβ1γ2 has a significantly higher affinity for the SNARE complex than Gβ1γ1 (10, 39). Gγ2 peptides identified in the array were mapped onto the Gβ1γ2 crystal structure (Fig. 1*E*, Gβ1 removed for simplicity). The Gγ2 N-terminal helix (H1) is shown in red, and the C-terminal loop (L1) is shown in orange.

Peptide binding data were used to generate tSNARE interaction heatmaps for each Gβ (Fig. 1*F*) and Gγ (Fig. 1*G*) isoform. These results suggest that Gβ1-4 interact with tSNARE within the same six regions (Fig. 1*F*). The peptides that interact with tSNARE were clustered near the N-terminal α-helix (H1), L1, and blades B1-B2 and B6-B7 of the β-propeller core (Fig. 1, *B* and *F*). These results are consistent with previous mutagenesis studies of Gβ1 (39). tSNARE showed decreased binding to Gβ5 peptides, but weaker interactions were detected in some of the same domains present in the Gβ1-4 subunits (Fig. 1*F*).

The N-terminal α-helix (H1) of nearly all Gγ isoforms was important for tSNARE interactions (Fig. 1*G*). We also observed tSNARE interactions near the C-terminus of Gγ-isoforms (L1). In some cases, the interaction is weak and encompasses only one peptide (Fig. 1*G*). Other isoforms show much stronger binding across larger regions of the C-terminus. This suggests that there may be more than one interaction site between Gγ and tSNARE, one at the N-terminus and the other near the C-terminus of Gγ. Recent work from our group has shown that Gβ1γ2 interacts with tSNARE complexes comprised of numerous syntaxin isoforms, SNAP23, and SNAP29 (46). Conceivably, different combinations of tSNAREs prefer to interact with different Gβγ subunits *via* alternate sites, providing an additional layer of specificity to the signaling cascade.

### Critical residues of G**β**1**γ**2-mediating interactions with tSNARE

**Figure 2:**
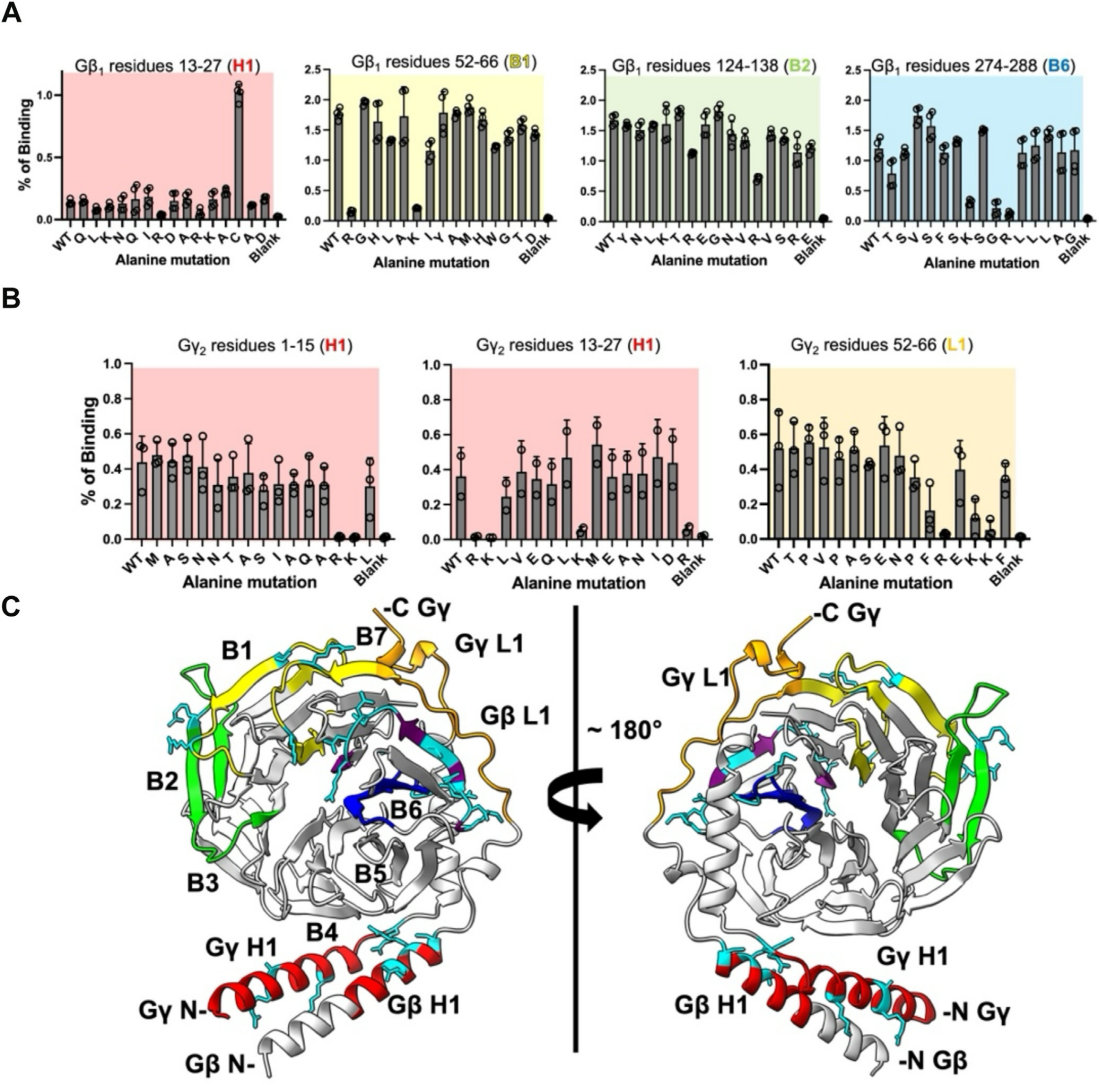
**Alanine-scanning mutagenesis screening of Gβγ peptides that bind tSNARE.***A and B*, peptide arrays with selected peptides from Gβ1 and Gγ2 containing single alanine substitutions at indicated positions in the wild-type sequence were prepared and probed with tSNARE. The first bar in each graph corresponds to the mean percent total densitometry of the wild-type peptide. The next 15 bars in each graph to the right are mutant peptides with a single alanine substitutions of the residues at indicated positions. *A*, Gβ1 peptide residues that showed a ≥50% reduction in tSNARE binding when substituted for Ala are: R19, R22, R52, K57, W82, K89, R96, R134, K280, G282, R283, G306, V307, L308, H311, and R314. Ala substitution of C25, C121, D303, and D312 resulted in an enhancement in tSNARE binding (n = 3–4 technical replicates). *B*, Gγ2 peptide residues identified are: R13, K14, K20, R27, K62, K64, and K65 (n = 2–3 technical replicates). Individual data points are shown superimposed onto bar graphs. *C*, the peptides identified by the array analysis as important for binding were mapped onto the Gβ1γ2 crystal structure (PDB: 6CRK, (45)) as colored in Figure 1. The residues whose alanine substitution resulted in significant loss of tSNARE binding to peptides are shown as cyan stick models. Bar graphs for each peptide tested are shown in Fig. S5.

To identify specific residues on Gβ1 and Gγ2 involved in tSNARE interactions, we subjected the peptides that bound tSNARE to alanine-scanning mutagenesis. For each peptide, the first spot was the wild-type sequence of a given peptide, followed by spots with a single alanine substitution. Figure 2 shows alanine scanning of Gβ1 and Gγ2 peptides with the position of the substituted residue indicated on the x-axis. The bar graphs represent the percent total binding of all peptides with loss (or gain) of binding in a mutant. Alanine substitutions of residues that exhibited significant loss (≤50% of WT) or gain (≥50% of WT) of tSNARE binding were mapped onto the crystal structure of Gβ1γ2 and shown as cyan stick models (Fig. 2*C*).

Gβ1 residues that showed significantly decreased tSNARE binding when substituted for Ala were: Arg19 and Arg22 from H1, Arg52, Lys57, Trp82, Lys89, and Arg96 from B1, Arg134 from B2, Lys280, Gly282, and Arg283 from B6, and Gly306, Val307, Leu308, His311, and Arg314 from B7 (Fig. 2, *A* and *C*). Interestingly, substitution of Gβ1 residues Cys25, Cys121, Asp303, and Asp312 for Ala significantly enhanced tSNARE binding (Fig. 2*A*, Fig. S5). However, Trp82, Gly282, and Cys121 are buried within the protein core according to the Gβ1γ2 crystal structure (45), and therefore, would not likely be accessible for tSNARE interactions upon folding. Critical Gγ2 residues are Arg13, Lys14, Lys20, and Arg27 from H1, and Lys62, Lys64, and Lys65 from L1 (Fig. 2, *B* and *C*). These results indicate the importance of basic residues on both Gβ1 and Gγ2 for tSNARE binding.

### G**γ**-derived peptides compete with full-length G**β**1**γ**2 for binding to SNAP25

**Figure 3:**
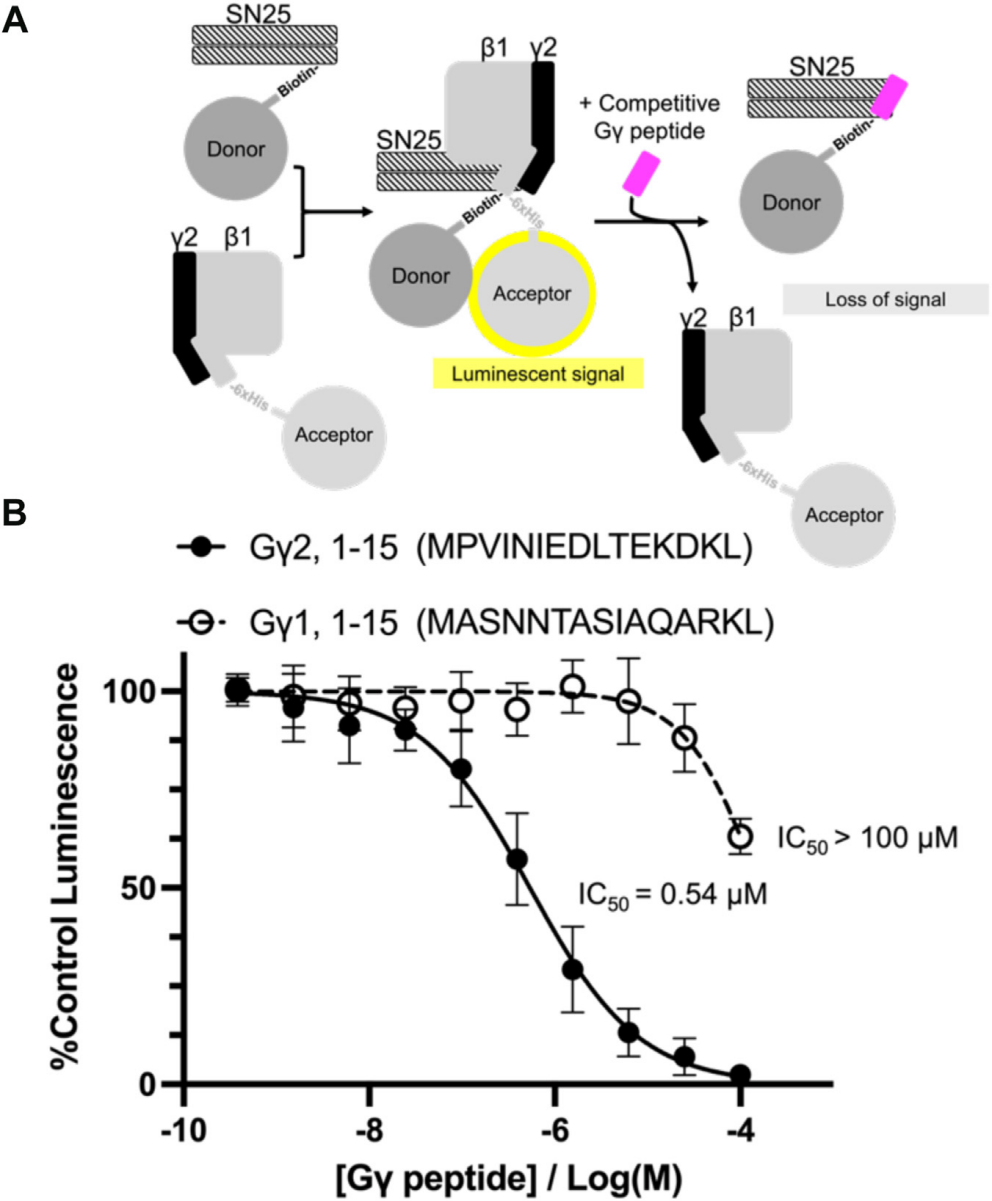
**Disruption of full-length Gβ1γ2/SNAP25 interactions by Gγ peptides identified in peptide array analysis.***A*, schematic illustrating assay principle. 20 nM biotinylated SNAP25 (*black stripes*) and 180 nM Gβ1γ2 (*gray, black*) was conjugated to Alphascreen donor and acceptor beads, respectively. Proximity of Alphascreen beads due to Gβ1γ2/SNAP25 interactions produces a luminescent signal. Competitive Gγ peptides (*pink*) bind to SNAP25 and displace Gβ1γ2, causing a concentration-dependent decrease in luminescence. *B*, competition-binding curves were generated for Gγ peptides that exhibited intense tSNARE binding in peptide array analysis experiments. A value of 100% was assigned to the average of all conditions tested containing only DMSO as a positive control. The data points correspond to the mean ± SD percentage of positive control luminescence (n = 3 technical replicates). Data were fitted with a four-parameter non-linear regression model in GraphPad Prism to obtain IC_50_ values for each peptide (Table 1). Curves for each peptide are shown in Fig. S6. Negative control experiments were performed by confirming that various concentrations of GST did not disrupt Gβ1γ2/SNAP25 interactions (Fig. S6).

**Figure 4:**
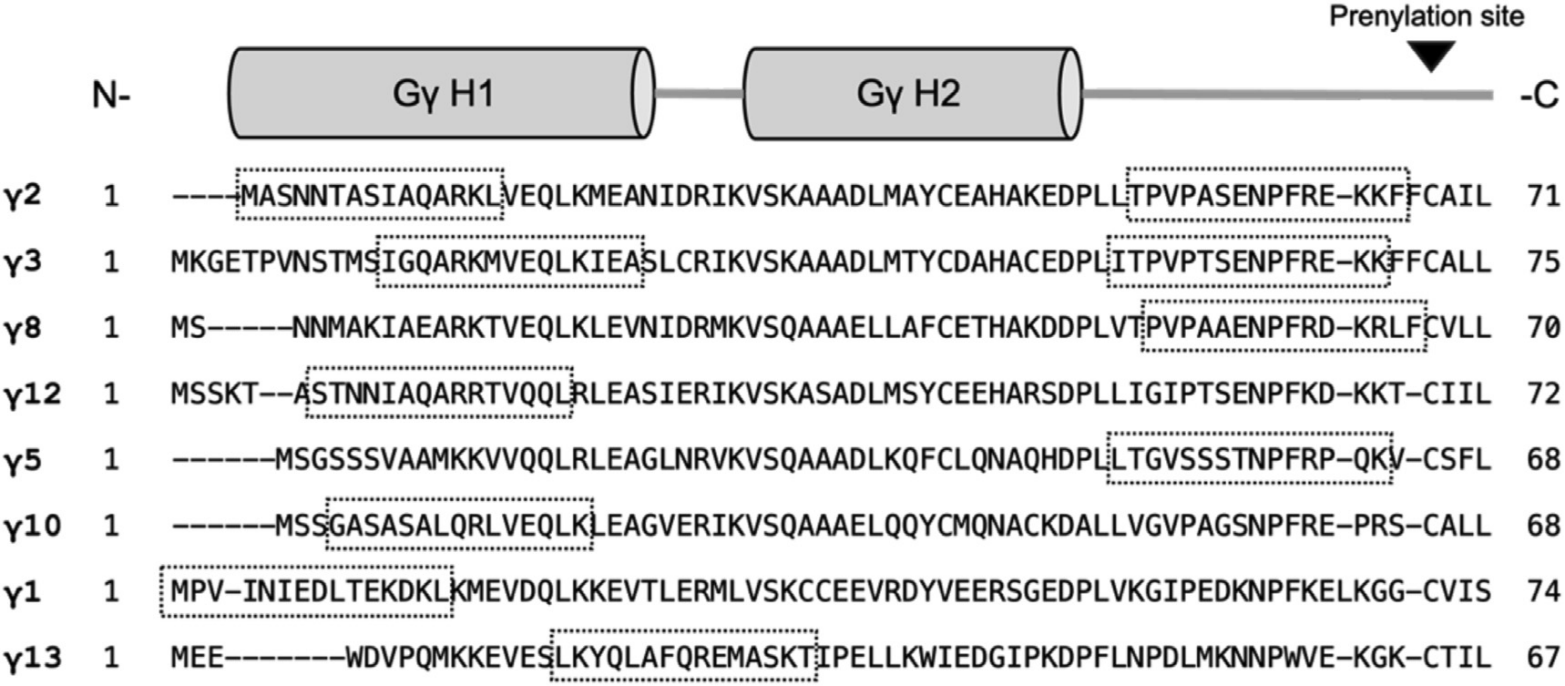
**Multiple sequence alignment of G**γ** isoforms tested in Gβ1**γ**2/SNAP25 competition binding assay.** Each isoform was aligned to Gγ2 using Clustal Omega. Regions corresponding to the sequences of tested peptides are outlined in dashed boxes. Secondary structural elements are shown as *gray* cylinders for reference.

Previous studies showed that the affinity of Gβ1γ1 is much lower than Gβ1γ2 for both monomeric SNAP25, tSNARE, and the ternary SNARE complex (10, 26, 39). This suggests that the primary sequence of the Gγ isoform contributes significantly to the interaction. To investigate this, we measured the ability of peptides derived from various Gγ isoforms to disrupt the interaction between full-length Gβ1γ2 and SNAP25 using the Alphascreen competition-binding assay (33, 39). We found that the Gγ2 N-terminal peptide was far more potent in inhibiting Gβ1γ2/SNAP25 interactions than that of Gγ1 (Fig. 3). This result is consistent with our peptide array analysis data (Fig. 2, *B* and *C*) as well as previous studies (10, 39). Using this method, we quantified IC_50_ values for peptides corresponding to other Gγ isoforms that interacted with tSNARE (Table 1, Fig. 4). These data show a clear preference of SNAP25 for the N-terminus of Gγ2 and Gγ10 as well as the C-terminus of Gγ8.

**Table 1:** Potencies of G**γ** peptides in inhibiting G**β**1**γ**2-SNAP25 interactions

G**γ** isoform	Peptide sequence	IC_50_ (μM)
Gγ2, res. 1–15	MASNNTASIAQARKL	0.54
Gγ2, res. 52–66	TPVPASENPFREKKF	12.62
Gγ3, res. 13–27	IGQARKMVEQLKIEA	4.04
Gγ3, res. 56–70	ITPVPTSENPFREKK	7.01
Gγ8, res. 52–66	PVPAAENPFRDKRLF	0.64
Gγ12, res. 7–21	STNNIAQARRTVQQL	2.91
Gγ5, res. 49–63	LTGVSSSTNPFRPQK	1.72
Gγ10, res. 4–18	GASASALQRLVEQLK	0.43
Gγ1, res. 1–15	MPVINIEDLTEKDKL	>100
Gγ13, res. 16–30	LKYQLAFQREMASKT	9.46

Competition between select Gγ peptides and full-length Gβ1γ2 for binding to SNAP25 was assessed using the Alphascreen competition-binding assay (n = 3 technical replicates).

**Figure 5:**
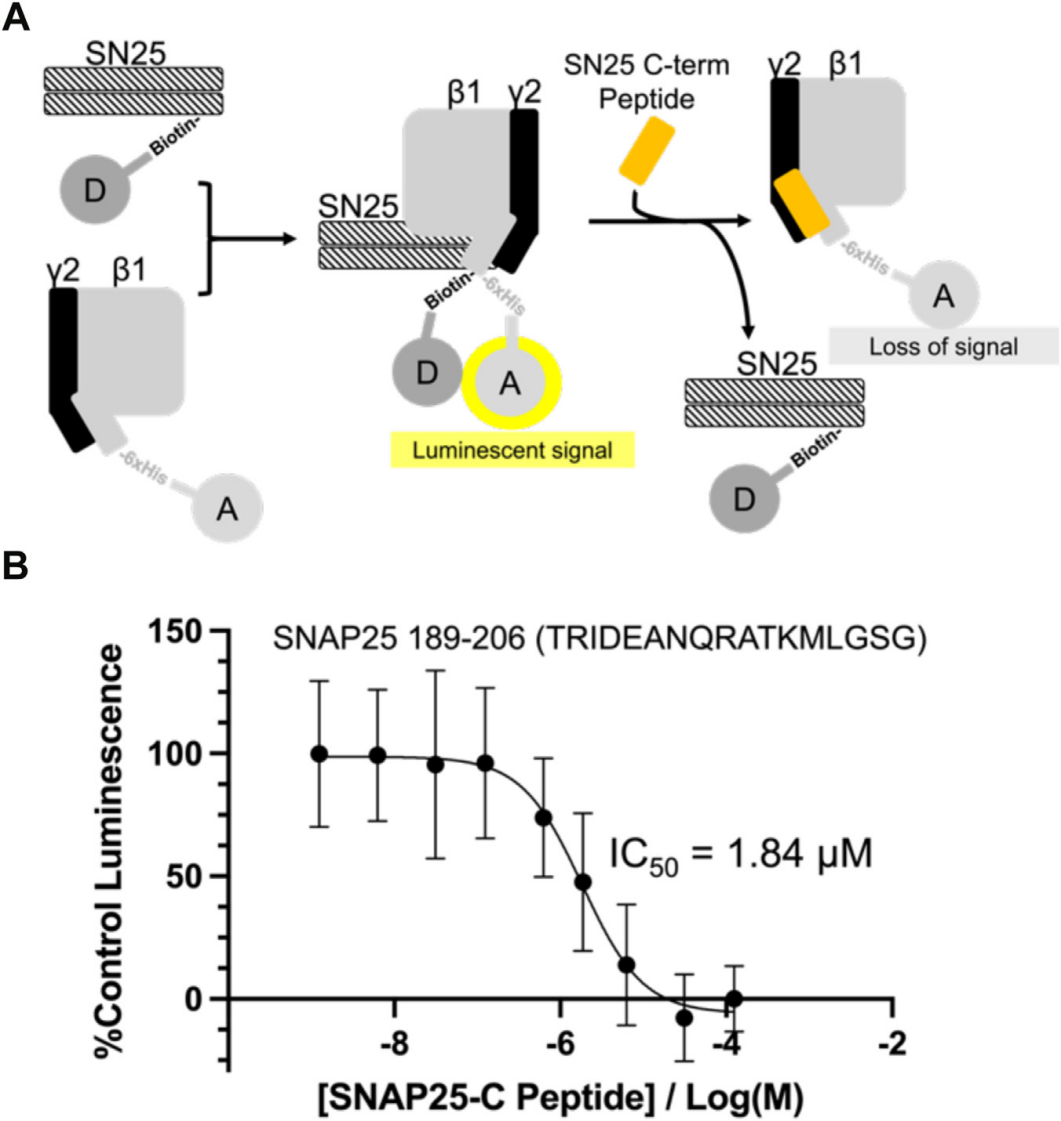
**The SNAP25 C-terminal peptide competes with full-length SNAP25 for binding to Gβ1*γ*2.***A*, diagram of the Alphascreen competition assay principle. The SNAP25 C-terminal peptide (residues 189–206, *orange*) displaces full-length SNAP25 (*black stripes*) from Gβ1γ2 (*gray/black*). *B*, competition binding curve of the C-terminal SNAP25 peptide (IC_50_ = 1.84 μM, 95% CI: 1.27–2.70 μM). Data points represent the mean ± SD percent positive control luminescence (n = 21 technical replicates).

Our group has also previously established the importance of the C-terminus of SNAP25 for Gβγ interactions (12, 33). We have recapitulated these results in this study by evaluating competition between the SNAP25 C-terminal peptide (residues 189–206) and full-length SNAP25 for binding to Gβ1γ2 (IC_50_ = 1.84 μM, 95% CI: 1.27–2.70 μM, Fig. 5). The ability of peptides to disrupt complex formation between SNAP25 and Gβ1γ2 indicates that the N-terminus of Gγ2 and the C-terminus of SNAP25 constitute an essential part of this protein–protein interaction.

### G**β**1**γ**2 has a higher affinity for the partially zipped pre-fusion SNARE mimetic than for the fully zipped mimetic

**Figure 6:**
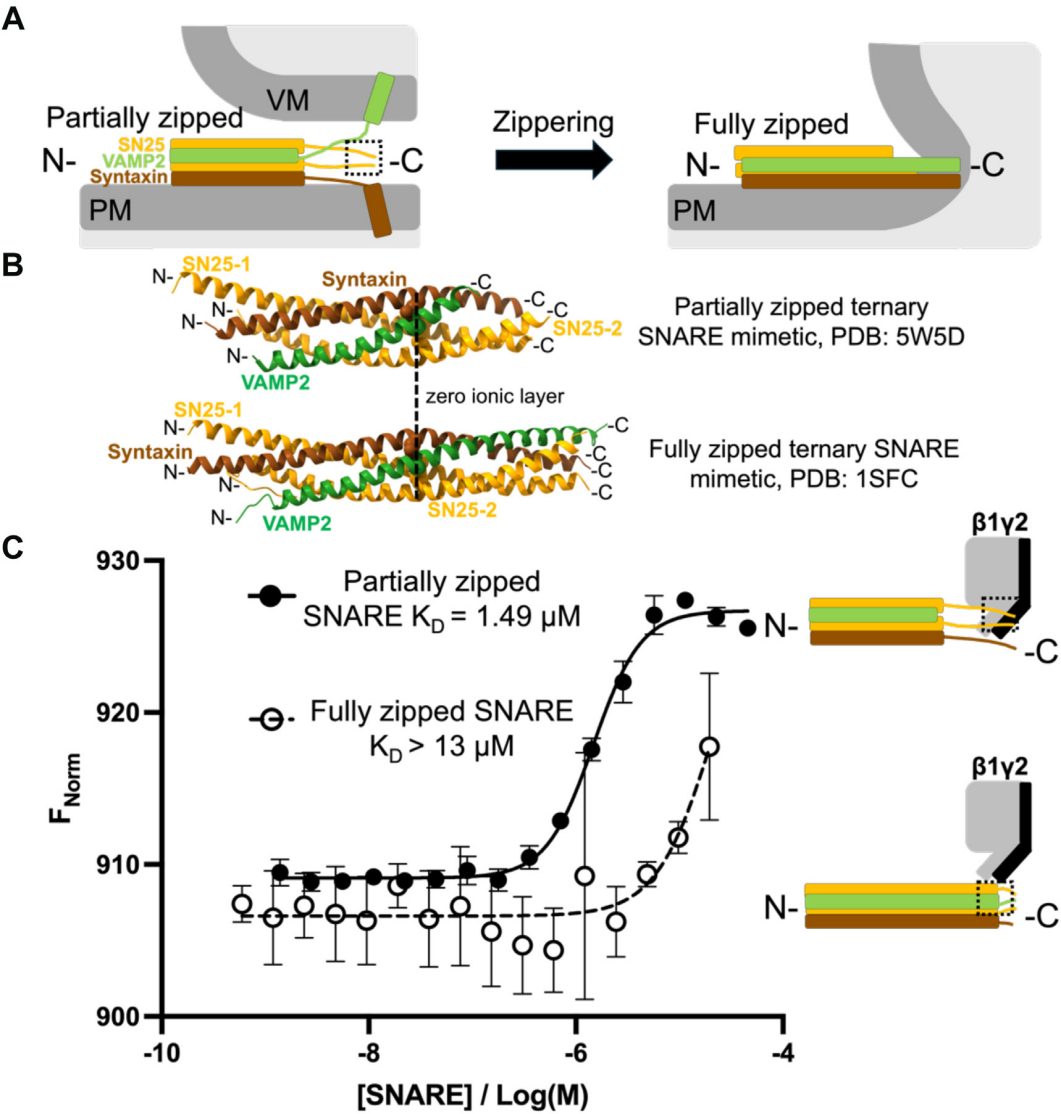
**Gβ1γ2 preferentially interacts with the partially zippered ternary SNARE mimetic.***A*, simplified diagram illustrating ternary SNARE in the partially zipped (*left*) and fully zipped (*right*) conformations. In the partially zipped pre-fusion conformation, the transmembrane helix of VAMP2 (*green*) is embedded in the synaptic vesicle membrane while the transmembrane helix of syntaxin-1a (*brown*) is inserted into the presynaptic membrane. The C-terminal SNARE domains remain disordered prior to fusion and are more sterically available for interactions with regulatory proteins. The critical Gβ1γ2 binding site located on the C-terminus of SNAP25 (*orange*) is outlined by the dashed box. N-to-C-terminal zippering of the complex orders each SNARE domain into the core helical bundle and provides the energy required for membrane fusion. *B*, crystal structures of the partially zipped (*top*, (41)) and fully zipped (*bottom*, (42)) ternary SNARE mimetics which contain only the SNARE domains of VAMP2, syntaxin1a, and SNAP25. The partially zipped SNARE contains a C-terminal truncation of the VAMP2 SNARE domain which traps the complex in a pre-fusion conformation. *C*, MST affinity analysis of 250 nM fluorescently labeled Gβ1γ2 binding to partially zipped (closed circles, K_*D*_ = 1.48 μM, 95% CI 1.33–1.64 μM) and fully zipped (open circles, K_*D*_ > 13 μM) soluble ternary SNARE mimetics (mean ± SD, n = 3 technical replicates). (*Inset*) We hypothesize that the Gβ1γ2 binding site is more accessible in the partially zipped conformation (*top*) than in the fully zipped conformation (*bottom*) thereby enhancing the affinity of the interaction.

After vesicle docking but before vesicle fusion, the SNARE complex exists in a partially zipped conformation in which the C-terminus of the complex is likely disordered (Fig. 6*A*, left) (41, 47, 48). The free energy released upon SNARE complex ordering into a four-helical bundle is thought to contribute significantly to the energy required for vesicle fusion (Fig. 6*A*, right) (48, 49, 50). Because Gβ1γ2-SNARE interactions occur late in the vesicle priming/docking cycle (11, 28), we hypothesized that Gβ1γ2 would have enhanced affinity for the partially zipped conformation. Furthermore, the C-terminus of SNAP25 is more accessible for interactions with other proteins in the partially zipped conformation.

To address this, we expressed and purified partially and fully zipped ternary SNARE mimetics and measured the affinity of each for fluorescently labeled Gβ1γ2 using Microscale Thermophoresis (MST). The partially zipped mimetic contains the SNARE domains of rat syntaxin-1A (brown, residues 191–256) and SNAP25 (orange, residues 7–83 and 141–206), as well as a portion of the synaptobrevin-2 SNARE domain (VAMP2, green, residues 28–66, Fig. 6*B*, top) (41). C-terminal truncation of the synaptobrevin-2 SNARE domain traps the complex in a partially zipped, *trans* conformation. The fully zipped mimetic contains the SNARE domain of rat syntaxin-1a, SNAP25 (residues 7–83 and 141–204) and synaptobrevin-2 (residues 28–89, Fig. 6*B*, bottom) (42, 43). We found that Gβ1γ2 binds to the partially zippered SNARE complex with a much higher affinity (K_*D*_ = 1.49 ± 0.15 μM, closed circles) than it does to the fully zippered complex (K_*D*_ > 13 μM, open circles Fig. 6*C*).

**Figure 7:**
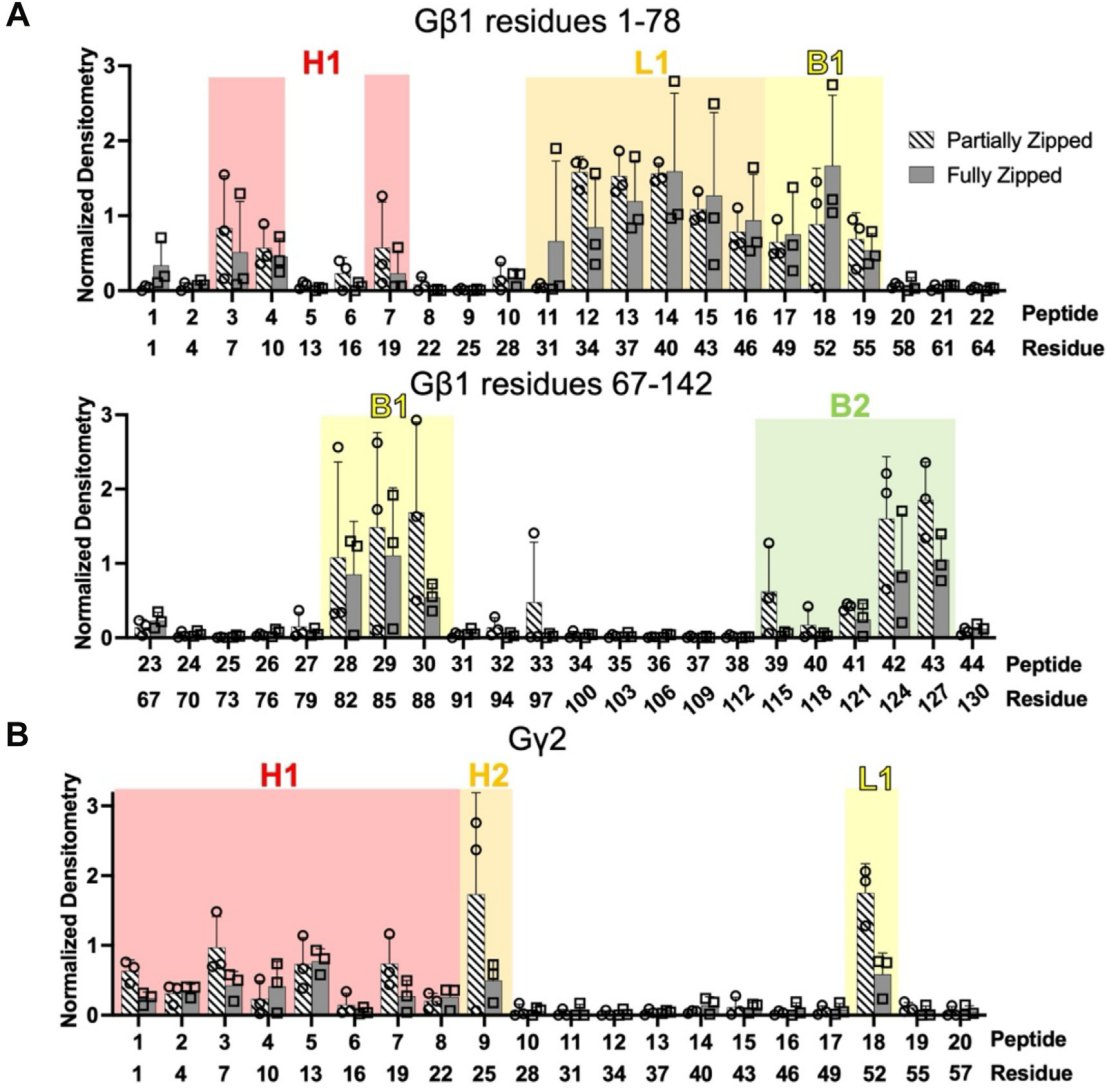
**Comparison of Gβ1γ2 binding patterns to ternary SNARE mimetics.** Quantification of (*A*) Gβ1 and (*B*) Gγ2 peptides binding to the partially zipped (*black striped bars*) and fully zipped SNARE (*gray bars*). Densitometry measurements were normalized to a positive control peptide spot corresponding to a portion of the rat complexin-1 central helix (residues 48–62). Bar graphs represent the mean ± SD of three technical replicates. *A*, both SNARE mimetics interact with the N-terminal α-helix (H1, *red*), L1 (*orange*), B1 (*yellow*), and B2 (*green*) of Gβ1. Only peptides 92 and 101 of B6 bound to both ternary SNAREs (Fig. S8), in contrast to tSNARE, which bound peptides 91 to 93 and 99 to 102 of B6 and B7 (Fig. 1*A*). *B*, both SNARE mimetics showed similar interactions with the N-terminal α-helix of Gγ2 (H1, *red*). Peptides nine and 18 (L1, orange) showed a statistically significant increase in binding to partially zipped SNARE (2-way ANOVA, *p* ≤ 0.0001).

To identify which regions of Gβ1γ2 contribute to the enhanced affinity for partially zipped SNARE, we performed an additional peptide array experiment in which we presented Gβ1γ2 peptides to both SNARE mimetics and probed membranes for SNAP25 (Fig. 7). Binding was quantified as a fraction of the densitometric signal of a positive control peptide corresponding to residues 48 to 62 of rat complexin-1, which interacts with the ternary SNARE complex in co-crystal structures (41, 51, 52).

Both partially zipped and fully zipped ternary SNARE mimetics bound to the same regions of Gβ1γ2 (Fig. 7). On Gβ1, binding was observed in the N-terminal α-helix (H1, red), the loop between H1 and the β-propeller domain (L1, orange), and blades B1 (yellow) and B2 (green) (Fig. 7*A*). The N-terminal α-helix of Gγ2 (H1, red), as well as the C-terminal loop (L1, orange), also interacted with both ternary SNARE mimetics (Fig. 7*B*). Two-way ANOVA statistical analysis with multiple comparisons revealed that the binding of Gγ2 peptides 9 and 18 was significantly higher to partially zipped SNARE than to fully zipped (*p* ≤ 0.0001, Fig. 7*B*). These results suggest that while the same residues of Gβ1γ2 are involved in binding both SNARE mimetics, the binding to partially zipped SNARE is enhanced.

The same peptides from Gγ2 bound to ternary SNARE (Fig. 7*B*) and tSNARE (Fig. 1*D*), except for peptide 19, which bound to tSNARE but not ternary SNARE. Gβ1 peptides also showed a similar binding pattern to tSNARE (Fig. 1*A*) and both ternary SNARE mimetics (Fig. 7*A*). Peptides 5 to 6 from the N-terminal α-helix of Gβ1 (H1) bound to tSNARE (Fig. 1*A*) but not ternary SNARE (Fig. 7*A*). Also, Gβ1 peptide 3 bound to both ternary SNARE mimetics but not to tSNARE. These findings indicate that the same general regions of Gβ1γ2 are involved in binding tSNARE, partially zipped ternary SNARE, and fully zipped ternary SNARE.

### Nanobody-5 (Nb5) blocks G**β**1**γ**2-SNAP25 interactions

**Figure 8:**
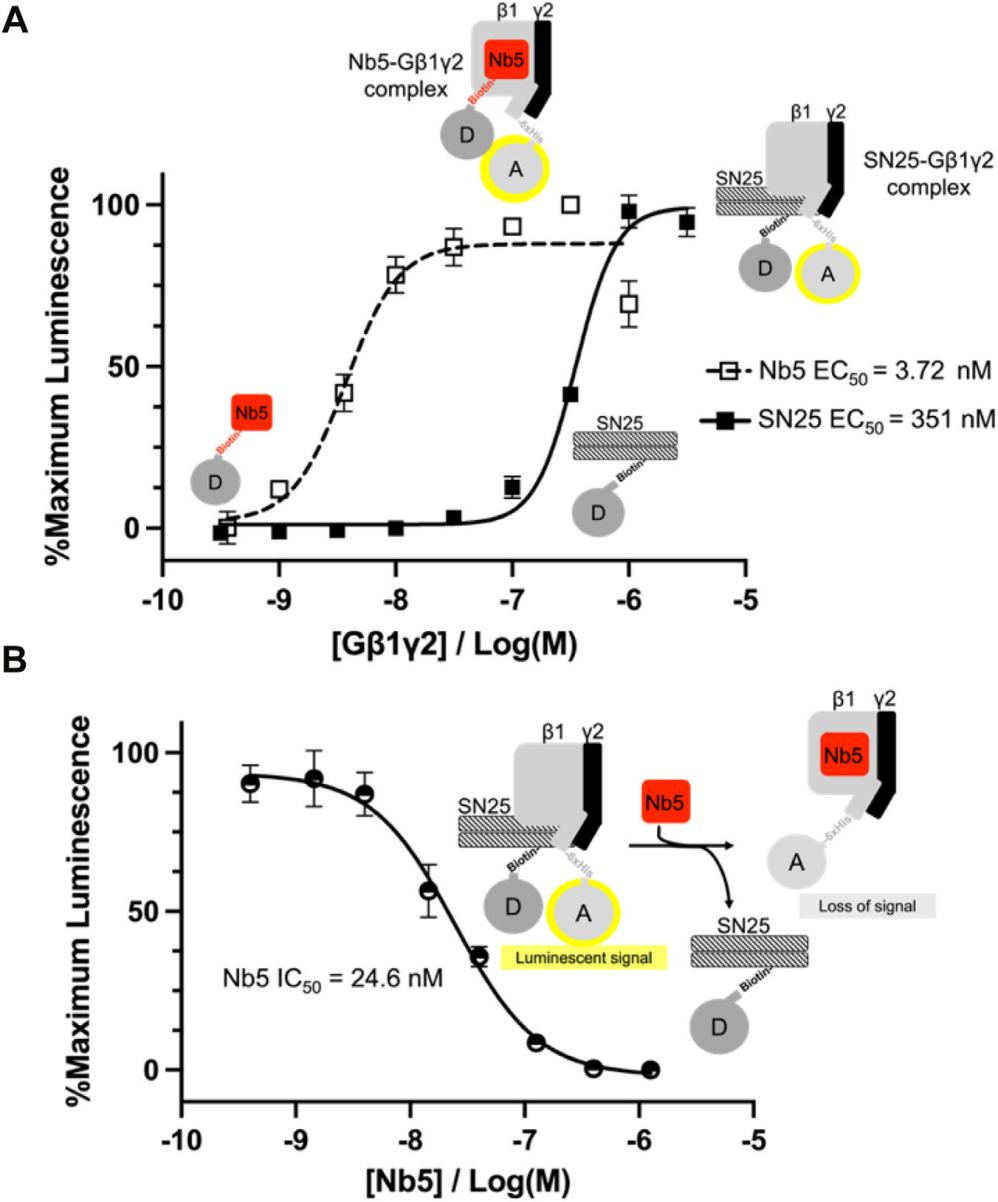
**Nb5 disrupts Gβ1γ2/SNAP25 interactions.***A***,** affinities of Gβ1γ2 for Nb5 (*red, open squares*) and SNAP25 (*orange, closed squares*) determined in the Alphascreen assay. Nb5 binds to the center of the Gβ1 β-propeller domain (53) and is not expected to hinder interactions involving the N-terminal coiled-coil domain. The affinities of Gβ1γ2 for Nb5 and SNAP25 were determined to be 3.72 nM (95% CI: 2.11–5.20 nM) and 351 nM (95% CI: 326–378 nM), respectively. *B*, the Alphascreen competition-binding assay was used to evaluate the effects of Nb5 concentration on Gβ1γ2/SNAP25 interactions. Nb5 is a potent inhibitor of the Gβ1γ2-SNAP25 complex (IC_50_ = 24.6 nM 95% CI: 19.2–31.7 nM). The means ± SD of three technical replicates are shown.

We also sought to test the ability of a Nanobody (Nb5) (53), which binds to the Gα interface of Gβ1, to compete with SNAP25 for binding to Gβ1γ2. Analysis of the Gβ1γ2-Nb5 crystal structure (53) showed that Nb5-Gβ1 interactions are localized in the center of the β-propeller domain. The presence of Nb5 would likely disrupt SNAP25 interactions involving the β-propeller domain of Gβ1, as several residues identified in our Ala scan are present at the Gβ1-Nb5 interface (PDB: 6B20) (53). However, it's unclear whether interactions involving the N-terminal coiled-coil would also be affected, as the Gγ1 is not involved at the interface. We tested whether Nb5 could block the Gβγ-SNAP25 interaction using the Alphascreen competition-binding assay. Figure 8 shows potent inhibition by Nb5 (IC_50_ = 25 nM), suggesting that Nb5 disrupts critical binding site(s) for SNAP25 on Gβ1γ2.

## Discussion

The selectivity between different Gβ and Gγ isoforms in forming Gβγ heterodimers has been established (54, 55, 56, 57), however, the consequences of this selectivity on the modulation of certain effectors remains unclear. In general, Gγ subunits may be classified as Gγ1-like or Gγ2-like. The first category includes Gγ1 and Gγ11, while the ten other Gγ subunits comprise the second category. A critical point of differentiation is found in the CaaX-box motif: the residue X is a serine in Gγ1-like subunits, enabling farnesyltransferase to farnesylate the C-terminus (13, 58). Gγ2-like subunits instead have a leucine residue in this position, conferring geranylgeranylation by geranylgeranyltransferase I instead. It has been previously determined that Gγ2-containing Gβγ subunits have substantially higher affinity for the SNARE complex and ability to inhibit exocytosis than Gγ1-containing Gβγ subunits (10, 26, 39). However, the molecular determinants underlying this difference in potency had not been illuminated. Here, we define the molecular interface between tSNARE and each Gβ and Gγ isoform and identify the residues critical for the Gβ1γ2-tSNARE interaction using peptide arrays (Fig. 1). We show that peptides from Gγ2-like isoforms can inhibit interactions between full-length Gβ1γ2 and SNAP25 with varying potencies, with N-terminal sequences within Gγ3 and Gγ12 providing minimal inhibition, while Gγ2, Gγ8_,_ and Gγ10 show six to sixteen-fold greater potency (Figs. 3 and 4). From this, we conclude that the primary sequence of the Gγ subunit contributes to the binding interface of Gβγ with SNARE, at odds with the notion that the identity of the prenyl group attached to the Gγ is the sole determinant of potency, reflecting more efficient membrane targeting.

This study, and previous studies produced by our group, highlights the importance of positively charged arginine and lysine residues on both Gγ and SNAP25 (12, 33). Despite this, a predominately positively charged binding surface for both effectors would be anticipated to be repulsive. The identity of the negatively charged residues required for the Gβγ-SNARE interaction are less well understood. Two such Gβγ-binding residues on SNAP25 are Glu62, located within the first of two helices of SNAP25 contributing to the four-helix bundle, and Asp99, located within the flexible linker region connecting the first and second helices (12). The lack of secondary structure in the region where Asp99 is located potentially allows the residue to interface with a series of positively charged residues identified in this data, including Gγ_2_ Arg13 and Lys14 (Fig. 2*B*). However, since the positively charged Lys102 on SNAP25 is also a key binding residue close to Asp99, this hypothesis predicts that a negatively charged residue has to be close to Arg13 and Lys14. Three such residues exist in proximity on the Gα-binding surface of Gβ, including Asp163, Asp186, and Asp205. On Gγ2, residue Glu17 is also in proximity; however, Ala substitution of Glu17 does not interfere with the binding of Gγ2 13 to 27 to tSNARE, suggesting it is not critical for the interaction (Fig. 2*B*). Interestingly, Ala substitution of Gβ1 residues Cys25, Asp303, and Asp312 resulted in enhanced tSNARE binding (Fig. 2*A*, Fig. S5). Interactions between SNAP25 and syntaxin within the tSNARE complex are largely comprised of hydrophobic packing of residues arranged in heptad repeats (42). Therefore, it is probable that hydrophobic packing interactions also contribute to Gβγ binding in addition to electrostatic interactions. Substitution of polar Cys and acidic Asp for Ala potentially allows for improved hydrophobic packing of these regions of Gβ1 with the hydrophobic tSNARE core. The limitations of the ResPep technique, including the limited secondary structure of the 15-mer peptides, render it unable to identify every residue of importance within a given protein-protein interaction.

We have identified two distinct hotspots for tSNARE and ternary SNARE binding to Gβγ: one encompassing the N-terminal coiled-coil motif, and the other involving blades B1-B2 and B6-B7 of the β-propeller domain (Fig. 1). One possible interpretation is that there could be a higher degree of stoichiometry between Gβγ and the SNARE complex, and the peptide array analyses have revealed two binding sites for SNARE on Gβ1γ2 (12, 39). Neurotransmitter release requires at least three intact SNARE complexes zippering simultaneously (59, 60). Therefore, it is conceivable that one Gβγ may interact with multiple SNAREs that are clustered near the fusing vesicle. The stoichiometry of the Gβγ-SNARE interaction represents an intriguing but not yet understood aspect of this inhibitory mechanism.

The importance of the N-terminal binding site is supported by the findings presented here as well as by previous findings, both in cell-based assays and *in vitro* binding studies (10, 39). Similarly, the importance of the β-propeller domain has also been demonstrated previously (10, 26, 39). For instance, Gαβγ heterotrimers cannot interact with SNAREs, suggesting that sequestering of the Gα-Gβ interface on the β-propellor blocks SNARE-interacting residues (26). Analysis of the heterotrimeric G-protein crystal structure reveals that the Gα-subunit hinders both the SNARE binding sites on the β-propeller and on the N-terminal coiled-coil of Gβγ (61). Furthermore, alanine substitution of Gβ1 residues Lys78 and Trp332, which are key for the Gα-Gβ interaction, enhances the potency of Gβγ-SNARE-mediated inhibition in PC12 cells (10). Peptide competition studies revealed that the peptide corresponding to Gβ1 residues 328 to 337 competed with full-length Gβ1γ2 for binding to SNAP25 with a potency of ∼28 μM (39). This is consistent with our findings that both the Gγ2 amino-terminal peptide and Nb5 are potent inhibitors of interactions between full-length Gβ1γ2 and SNAP25 (Figs. 3 and 8).

Interestingly, Gβ1γ2 has an enhanced affinity for the partially zipped SNARE complex (Fig. 6), yet the same general regions of Gβ1γ2 interact with both the partially and the fully zipped ternary SNARE complex (Fig. 7). We hypothesize that incorporation of synaptobrevin into the SNARE complex upon zippering reduces the availability of the C-terminal SNARE hydrophobic layers to Gβγ, but not the solvent exposed residues. The same regions of Gβ1γ2 bind to these solvent exposed residues, but the affinity is decreased due to the loss of hydrophobic packing contributions to the Gβγ-SNARE interaction. The result that Gβ1γ2 has an enhanced affinity for ternary SNARE in the partially zipped, pre-fusion conformation (Fig. 6) is consistent with previous *in-vivo* experiments demonstrating that Gβγ acts on the readily releasable pool of vesicles, in which the SNARE complex is docked and primed (11). Vesicle destaining experiments with FM dyes indicate that Gβγ interactions with the SNARE complex alter the stability of the fusion pore, and that Gβγ permits transient fusion but prevents full collapse of the vesicle (28). Therefore, we conclude that Gβγ binds to pre-formed SNARE complexes and sterically blocks the zippering of the SNARE complex required for full vesicle fusion. This interaction may also disrupt interactions between the SNARE complex and other regulatory proteins. For example, previous studies have established that Gβγ competes with the Ca^2+^ sensor synaptotagmin for binding to the SNARE complex in a Ca^2+^-dependent manner (26, 39). The exact details and consequences of Gβγ binding on SNARE complex conformation, and subsequent effects on SNARE interactions with other regulatory proteins, await structural determination of the Gβγ-SNARE complex.

**Figure 9:**
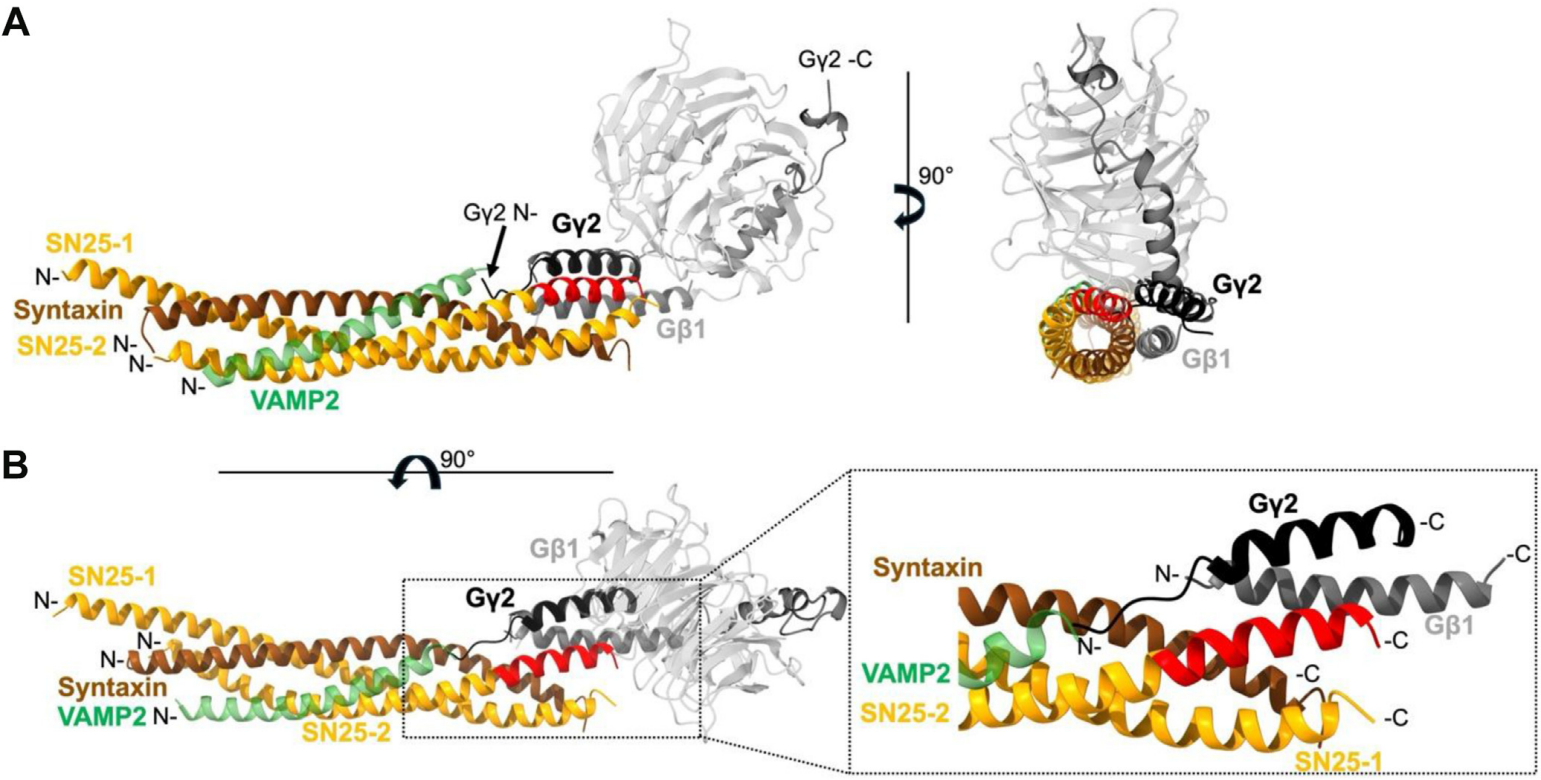
**Structural prediction of tSNARE bound to Gβ1γ2 N-terminal peptides**. Sequences for the SNARE domains of rat syntaxin-1a (residues 191–256, *brown*), and SNAP25 (residues 7–83 and 141–206, *orange*) were used to predict the structure of tSNARE bound to human Gβ1 (residues 1–25, *gray*) and Gγ2 (residues 1–24, *black*) using Chai-1. Rosetta was used for docking/redocking. *A*, view of the structural prediction with the crystal structures of Gβ1γ2 (PDB: 6CRK) and partially zipped ternary SNARE (PDB: 5W5D) superimposed onto the model as transparent cartoons for reference. (*left*) The N-terminal Gβ1γ2 coiled-coil is predicted to insert into the SNARE complex helical bundle near the C-terminus. (*right*) View of the predicted structure down the SNARE helical bundle which shows an interface between Gβ1γ2 and the C-terminal SNAP25 peptide (indicated in *red*). *B*, Close-up of the predicted interface with the critical SNAP25 C-terminal peptide.

Based on our findings that the N-terminal coiled-coil of Gβ1γ2 is an important site for interactions with tSNARE (Figs. 1 and 2), SNAP25 (Fig. 3), and ternary SNARE (Fig. 7), we used Chai-1 and Rosetta redocking/relaxation (62, 63, 64, 65, 66, 67, 68) to predict the structures of the Gβ1γ2 N-terminal peptides (Gβ1 residues 1–25, gray, Gγ2 residues 1–24, black) bound to tSNARE (Fig. 9). The crystal structures of Gβ1γ2 (45) and partially zipped SNARE (41) are shown superimposed onto the prediction as transparent cartoon models for reference. The Gβ1γ2 N-terminal coiled-coil is predicted to dock at the extreme C-terminus of the SNARE complex and insert into the SNARE helical bundle. This is a plausible binding pose in agreement with the data presented here as well as previous studies. The C-terminal peptide of SNAP25 (residues 189–206, indicated in red) is predicted to form an interface with both the Gβ1 (gray) and Gγ2 (black) N-terminal peptides (Fig. 9). Crystallographic studies of the partially zipped SNARE complex suggest that the C-terminal portion of SNAP25 and syntaxin are disordered prior to full incorporation of synaptobrevin into the core SNARE helical bundle (41), therefore, the prediction may overestimate the rigidity of this region. The predicted interfaces between the Gβ1γ2 peptides and the SNARE complex provide hypotheses that can be tested *via* mutagenesis to further define the molecular details of the interaction. Based on our collective findings, we propose that the N-terminal coiled coil domain of specific Gβγ isoforms stabilize the disordered C-terminus of the partially zipped SNARE complex, disrupting N-to-C-terminal incorporation of synaptobrevin, thereby inhibiting vesicle fusion. The precise role of the β-propeller of Gβ with the SNARE complex awaits further investigation.

## Conclusions

The interaction of the G-protein βγ dimer with the SNARE complex is an important regulatory mechanism inhibiting exocytosis. Here we probed the regions of Gβγ important for its binding to the SNARE complex. Systematic peptide screening revealed two regions, consistent across the family of 5 Gβ and 12 Gγ proteins, that bind to tSNARE. These two regions, the Gβγ coiled-coil domain and the Gβ propeller region, are spatially separated and may represent two SNARE complex binding sites. Increased access to critical residues at the C-terminus of SNAP25 in the partially zipped ternary SNARE complex likely drives the enhanced affinity for Gβ1γ2. Our results suggest that Gβγ disrupts SNARE complex zippering by steric hindrance of synaptobrevin binding, thereby inhibiting vesicle fusion. These data provide a roadmap for future experiments to further elucidate the molecular details of the interface.

## Experimental procedures

*Materials* Primary antibodies for SNAP25 (SC-376713, SC-136267) were purchased from Santa Cruz Biotechnology. HRP-conjugated secondary anti-mouse antibody (catalog #55220–0341) was purchased from Seracare. Enhanced chemiluminescence substrate (catalog #34580) was from ThermoFisher. FMOC amino acid derivatives were purchased from 21st Century Biochemicals. Alphascreen measurements were performed using the Alphascreen 6His Nickel Chelate Detection Kit purchased from Revvity (part #6760619C). For MST experiments, His-tagged Gβ1γ2 was fluorescently labeled using the Monolith Series His-Tag Labeling Kit—Red-tris-NTA 2nd generation from Nanotemper Technologies (SKU: MO-L018). Measurements were performed in Monolith standard treated capillaries also from Nanotemper Technologies (SKU: MO-K022).

### Protein purification

Wildtype tSNARE and SNAP25 were expressed and purified as described (12, 39). Plasmids encoding rat His-tagged synaptobrevin-2 (residues 28–89) and syntaxin-1A (residues 191–256) in a pACYDuet-1 vector was received as a gift from the laboratory of Dr Qiangjun Zhou (Addgene plasmid #70055). The plasmid encoding His-tagged synaptobrevin-2 (residues 28–66) and syntaxin (residues 191–256 in a pACYDuet-1 vector was purchased from Addgene (plasmid #106414). The plasmid encoding rat SNAP-25b (residues 141–206 and 7–83) was subcloned into a pETDuet-1 by GenScript. SNARE-containing plasmids were co-transformed into BL21 *E. Coli*. The SNARE complex was expressed and purified as described previously (41, 43). His-tagged soluble Gβ1γ2 (C68S) was expressed and purified from Sf9 cells as previously described (69). The plasmid encoding His-tagged Nb5 was received as a gift from the laboratory of Dr Krzysztof Palczewski. Nb5 was expressed and purified as described previously (53).

### Peptide Array analysis and far-Western blot

Peptide array synthesis was performed using the ResPep SL peptide synthesizer (Intavis AG) according to standard SPOT synthesis protocols (44, 70). Indicated peptides containing 15 amino acids were directly coupled to membranes *via* the C-terminus during synthesis. Dried membranes with peptides were soaked in 100% ethanol for 5 min then rehydrated in water (twice 5 min). The membranes were blocked for 1 h in Tris-buffered saline (TBS) with 5% milk and 0.05% Tween 20 (Sigma-Aldrich) and washed 3 times (5 min each) in TBS with 0.05% Tween 20 (TBS-T). The membranes were incubated overnight at 4 °C with each SNARE protein at a final concentration of 0.5 μM in 20 mM HEPES, pH 7.5 buffer containing 150 mM NaCl and 1 mM DTT. The buffer used for tSNARE experiments also contained 0.01% OG. The next morning, membranes were washed 3 times (5 min each) in TBS-T buffer and incubated with primary antibody (SNAP25) at 1:1000 dilution in TBS-T for 1 h. Spots were detected using HRP-conjugated secondary antibodies as described by the manufacturer. For densitometric analysis comparing Gβ and Gγ isoforms, the signal in individual dots was quantified as a percentage of total density detected on the membrane (Quantity One and Image Lab, Bio Rad, and Image J [Schneider *et al.*, 2012]). This allowed for comparison of intensities across all peptide experiments. Quantification of Gβ1 and Gγ2 peptide spots binding to ternary SNARE mimetics was performed by normalizing within each blot to a positive control corresponding to residues 48 to 62 of complexin-1. Statistical analysis was performed using one-way ANOVA followed by Dunnett's *post hoc* test using GraphPad Prism 8.0.

### Alphascreen competition-binding assays

Alphascreen luminescence measurements were performed as described (33). For peptide competition experiments, biotinylated SNAP25 was diluted to a 5 concentration of 100 nM in assay buffer (20 mM HEPES, pH 7.0, 10 mM NaCl, 40 mM KCl, 5% glycerol, and 0.01% Triton X-100). An EC_80_ concentration of 180 nM purified His6-Gβ1γ2 was made in assay buffer. Peptide stocks in DMSO were spotted onto 384- well white OptiPlates (PerkinElmer Life Sciences) at concentration ranges of 1 nM to 100 μM using a Labcyte Echo 555 Omics acoustic liquid handler (Labcyte), with DMSO being back-added to a final concentration of 0.1%. 4 μl of Gβ1γ2 solution was incubated with peptide for 5 min while shaking. After 5 min, 1 μl of biotinylated SNAP25 was added to a final concentration of 20 nM. Subsequent to incubation while shaking for an additional 5 min, 10 μl of Alphascreen Histidine Detection Kit (nickel chelate) acceptor beads were added to a final concentration of 20 μg/ml in assay buffer. The assay plate was agitated in dim green light for 30 min. At that point, Alphascreen Streptavidin Donor Beads were added to a final concentration of 20 μg/ml in dim green light. All aqueous solutions in this assay were manipulated by a Velocity 11 Bravo liquid handler (Agilent Automation Solutions). The final volume in the assay plate was 25 μl. After being spun down briefly to settle all fluid at the bottom of the well, plates were incubated for an additional 1 h at 27 °C before being read in the EnSpire. 20 nM biotinylated recombinant glutathione S-transferase in place of SNAP25 with Gβ1γ2 was used as a negative control for nonspecific binding in each assay (Fig. S6). IC_50_ concentrations for each peptide were determined by sigmoidal dose-response curve-fitting with variable slope.

For Nb5 binding experiments, 20 nM biotinylated Nb5 was incubated with varying concentrations of His-tagged Gβ1γ2 (0.1 nM - 1 μM) in a 384-well white Optiplate. Alphascreen acceptor beads were added to each well to a final concentration of 20 μg/ml and incubated for 30 min. 20 μg/ml Alphascreen donor beads were then added prior to incubation in a plate shaker for 1 h. Plates were read in a Biotek Synergy Neo. Concentration response curves were analyzed in GraphPad prism to derive EC_50_ values. For Nb5 competition experiments, biotinylated SNAP25 was reacted with an EC_80_ concentration of His-tagged Gβ1γ2 as described above. Nb5 was added in concentrations ranging from 0.4 nM to 1.26 μM.

### Microscale Thermophoresis

4 μM His-tagged Gβ1γ2 was labeled with Red-Tris-NTA using the Monolith series His-tag labeling kit protocol (Nanotemper Technologies). Concentration–response curves for purified partially zipped SNARE (1.38 nM–45.5 μM) and fully zipped SNARE (0.56 nM−19.5 μM) were then added to 250 nM fluorescently labeled Gβ1γ2 in PCR tubes. Samples were transferred to Monolith standard coated capillaries and read on a Nanotemper Monolith instrument.

## Data availability

All data described are contained within the manuscript. Representative raw peptide array images are available in the supplemental information.

## Supporting information

This article contains supporting information.

## Conflict of interest

The authors declare that they have no conflicts of interest with the contents of this article.
