# Supplementary material for: Molecular basis for Gβγ-SNARE-mediated inhibition of synaptic vesicle fusion
Source: J Biol Chem. 2025 Jun 14;301(8):110377. doi: 10.1016/j.jbc.2025.110377 (PMC12302718; doi:10.1016/j.jbc.2025.110377)
Supplement: Supporting information [file mmc1.docx]

**Molecular basis for Gβγ-SNARE mediated inhibition of synaptic vesicle fusion.**

**Supplemental Information.**

**Authors:** Anna R. Eitel^1^, Benjamin K. Mueller,^2^ Ali I. Kaya,^3^ Montana Young,^3^ Jackson B. Cassada,^3^ Eric W. Bell,^2^ Lauren Schnitkey,^3^ Zack Zurawski,^3^ Yun Y. Yim,^3^ Qiangjun Zhou,^4^ Jens Meiler,^2,5^ and Heidi E. Hamm^1,3^

^1^Department of Biochemistry, Vanderbilt University, Nashville, Tennessee 37232, USA

^2^Department of Chemistry, Vanderbilt University, Nashville, Tennessee 37235, USA

^3^Department of Pharmacology, Vanderbilt University, Nashville, Tennessee 37232, USA

^4^Department of Cell and Developmental Biology, Center for Structural Biology, Vanderbilt Brain Institute, Vanderbilt Kennedy Center, Nashville, Tennessee 37232, USA

^5^Institute for Drug Development, Leipzig University, Leipzig, Germany

*Included material:*

- Representative images of peptide array blots used for quantification in Figs. 1-2, and 7.
- Quantification of peptide blots for Gβ2-5 and Gγ3-13 used to generate heatmaps in Fig. 1.
- Quantification of Ala scan peptide arrays.
- Alphascreen competition binding curves used to calculate the EC_50_ values given in Table 1.


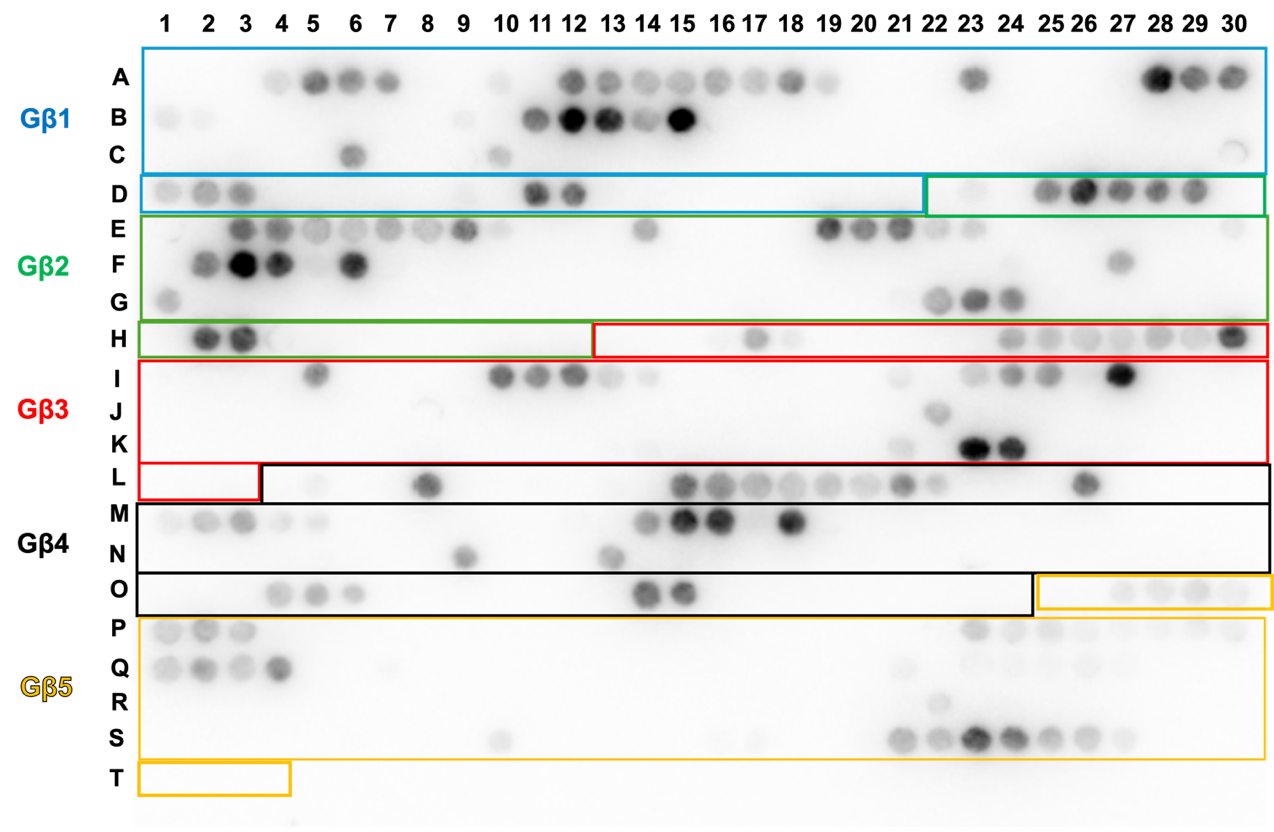


**S-1**. Representative blots showing peptide array analysis of (**A**) Gβ1-5 and (**B**) Gγ1-5 and Gγ7-13 binding to purified 0.5 μM tSNARE (there is no human Gγ6 isoform). Positions of peptides in each array are identified by row letter and column number. (**A**) Gβ1 peptides 1-110: A1-D20, (outlined in blue), Gβ2 peptides 1-110: D22-H11 (outlined in green), Gβ3 peptides 1-110: H13-L2 (outlined in red), Gβ4 peptides 1-110: L4-O23 (outlined in black), and Gβ5 peptides 1-128: O25-T2 (outlined in orange). The following positions in were left unspotted (blank): D21, L3, and T3. Positions H12, O24, and T4 contain Gly 15-mer peptides. (B) Gγ1 peptides 1-21: A1-A21 (outlined in black), Gγ2 peptides 1-20: A23-B12 (outlined in blue), Gγ3 peptides 1-21: B14-C4 (outlined in red), Gγ4 peptides 1-21: C6-C26 (outlined in green), Gγ5 peptides 1-19: C28-D16 (outlined in orange), Gγ7 peptides 1-19: D18-E6 (outlined in black), Gγ8 peptides 1-20: E8-E27 (outlined in blue), Gγ9 peptides 1-19: E29-F17 (outlined in red), Gγ10 peptides 1-19: F19-G7 (outlined in green), Gγ11 peptides 1-21: G9-G29 (outlined in orange), Gγ12 peptides 1-20: H1-H20 (outlined in black), and Gγ13 peptides 1-20: H22-I10 (outlined in blue). Positions A22, C27, E7, F18, G30, and I11 were not spotted with peptide. Positions B13, C5, D17, E28, G8, H21, and I12 were spotted with a Gly15-mer peptide.

**A**


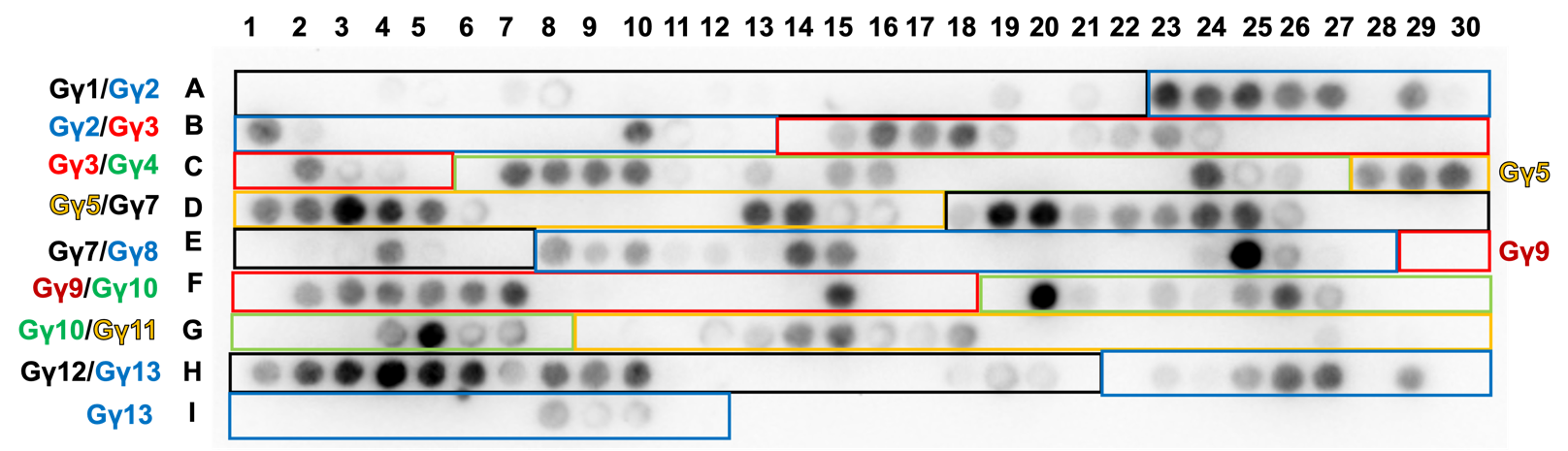


**B**


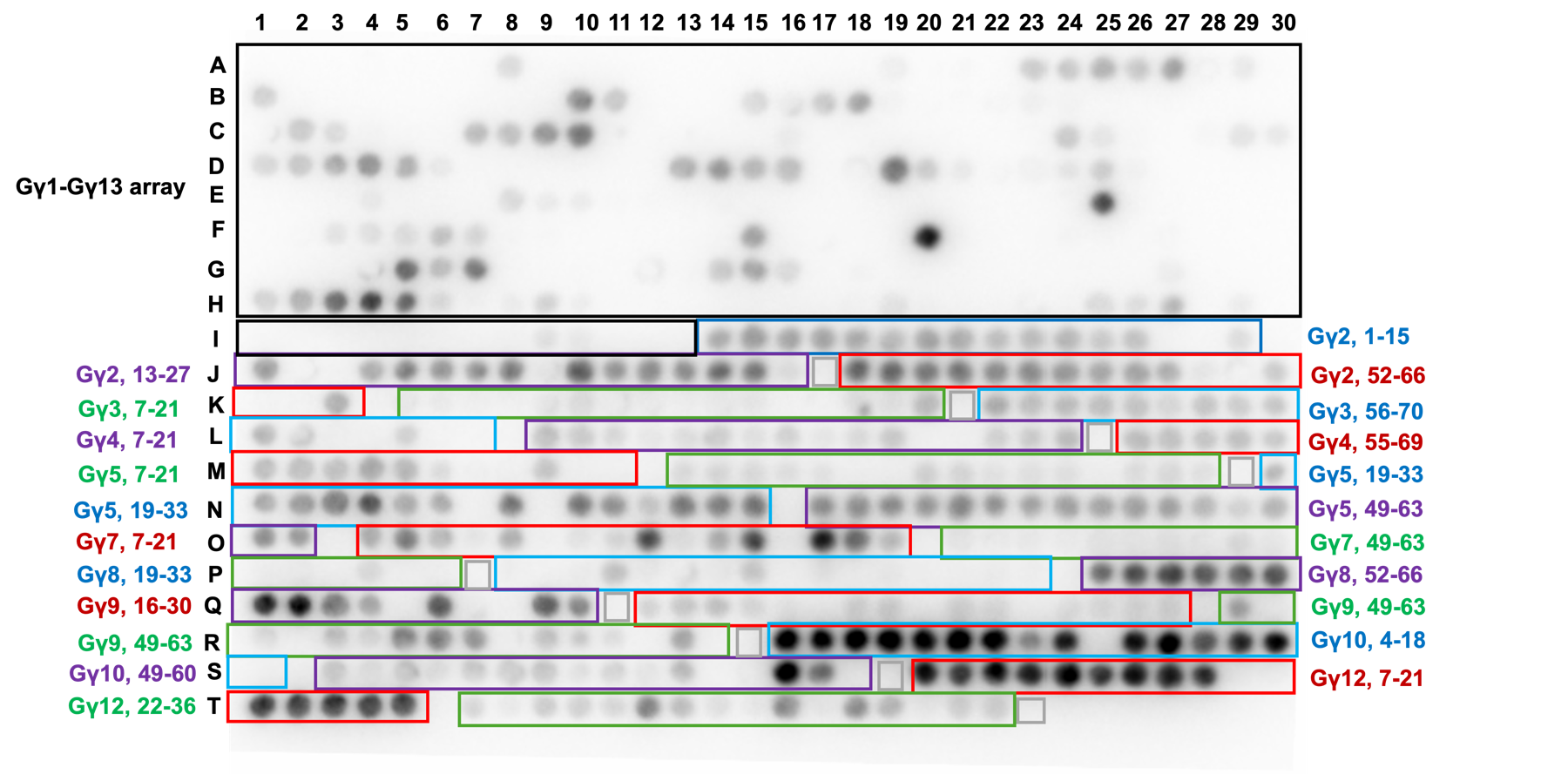


**S-2**. Representative Ala scan membrane array. Positions in the array are identified by row letter and column number. The first half of the sheet (A1-I13) contains a regular peptide array of Gγ isoforms (outlined in black). Ala scanned peptides are outlined in blue, purple, red, and green. The first peptide in each Ala scan corresponds to the WT 15-mer sequence, and each successive peptide spot contains a single Ala substitution at each position in the sequence. Gγ2 residues 1-15: I14-I29 (blue), Gγ2 residues 13-27: J1-J16 (purple), Gγ2 residues 52-66: J18-K3 (red), Gγ3 residues 7-21: K5-K20 (green) Gγ3 residues 56-70: K22-L7 (blue), Gγ4 residues 7-21: L10-L24 (purple) Gγ4 residues 55-69: L26-M11 (red), Gγ5 residues 7-21: M13-M28 (green), Gγ5 residues 19-33: M30-N15 (blue), Gγ5 residues 49-63: N17-O2 (purple), Gγ7 residues 7-21: O4-O19 (red), Gγ7 residues 49-63: O21-P6 (green), Gγ8 residues 19-33: P8-P23 (blue), Gγ8 residues 52-66: P25-Q10 (purple), Gγ9 residues 16-30: Q12-Q27 (red), Gγ9 residues 49-63: Q29-R14 (green), Gγ10 residues 4-18: R16-S1 (blue) Gγ10 residues 49-60: S3-S18 (purple), Gγ12 residues 7-21: S21-T5 (red), Gγ12 residues 22-35: T7-T22 (green).

**S-3**. Quantification of peptide array of Gβ isoforms. % of binding was quantified as the percent densitometry contributed per spot to the total densitometry detected on the membrane (mean ± SD, n = 3).

**S-4**. Quantification of peptide array of Gγ isoforms. % of binding was quantified as the percent densitometry contributed per spot to the total densitometry detected on the membrane (mean ± SD, n = 3).

**S-5**. Quantification of Gβ1 peptide alanine scan. % of binding was quantified as the percent densitometry contributed per spot to the total densitometry detected on the membrane (mean ± SD, n = 3). Ala substitutions of residues that caused significant loss (≤50% of WT) or gain (≥50% of WT) in the % binding of the peptide were mapped onto the crystal structure in Fig. 2C.

**S-6**. Alphascreen competition binding curves for Gγ 15-mer peptides (mean ± SD, n = 3). The ability of GST to disrupt Gβ1γ2/SNAP25 interactions was also tested as a negative control.


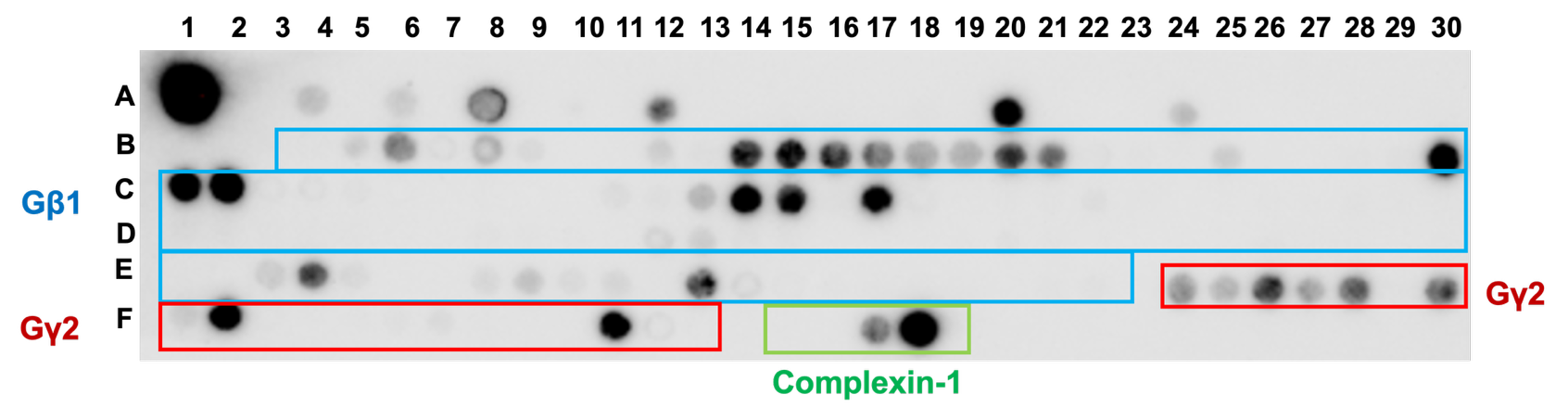

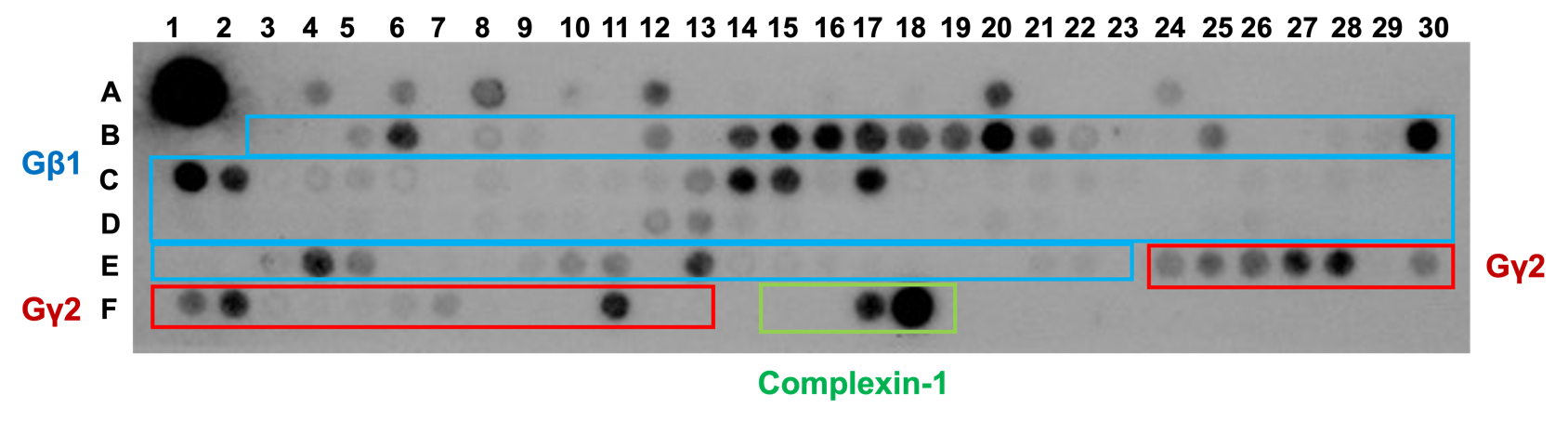


**S-7**. Representative blots for Gβ1γ2 peptides binding to **(A)** fully zipped and **(B)** partially zipped ternary SNARE mimetics. Rows of the array are labeled A-F and columns are labeled 1-30. Gβ1 peptides 1-110 are outlined in blue (B3-E22), Gγ2 peptides 1-20 are outlined in red (E24-F13), and complexin-1 peptides 1-4 are outlined in green (F15-F18). Densitometry for each peptide was normalized to complexin peptide 3 (F17, residues 48-62). The positive control for the SNAP25 primary antibody was spotted on A1 (rat SNAP25b residues 8-22). The control peptides in row A correspond to the following sequences: A4: Ala 15mer, A6: Gly 15mer, A8: Arg15-mer, A10: Glu15-mer, A12: Gβ peptide 4 scrambled sequence (most hydrophilic), A14: Gβ1 peptide 38 scrambled sequence (most hydrophobic), A16: Gβ1 peptide 1 scramble, A18: Gβ1 peptide 110 scramble, A20: Gγ2 peptide 18 scramble (most hydrophilic), A22: Gγ2 peptide 10 scramble (most hydrophobic), A24: Gγ2 peptide 1 scramble, and A26: Gγ2 peptide 20 scramble. To correct for differences in background, the signal corresponding to blank spots were subtracted from the signal from each peptide-containing spot prior to normalization.

A

B

**S-8**. Quantification of Gβ1 peptides 45-110 binding to the partially zipped (black striped bars) and fully zipped ternary SNARE (gray bars) mimetic.
